# Multi-Infection Patterns and Co-infection Preference of 27 Human Papillomavirus Types Among 137,943 Gynecological Outpatients Across China

**DOI:** 10.3389/fonc.2020.00449

**Published:** 2020-04-07

**Authors:** Guangdong Liao, Xiyi Jiang, Bin She, Huijuan Tang, Zhongyong Wang, Hongrong Zhou, Yan Ma, Weidong Xu, Hongxing Xu, Wen Chen, Jianguang Ji, Mingrong Xi, Tianhui Chen

**Affiliations:** ^1^Department of Gynecology and Obstetrics, West China Second University Hospital, Sichuan University, Chengdu, China; ^2^Key Laboratory of Birth Defects and Related Diseases of Women and Children Affiliated to the Ministry of Education, Sichuan University, Chengdu, China; ^3^Institute of Occupational Diseases, Hangzhou Medical College, Hangzhou, China; ^4^Department of Academic Development, Tellgen Corporation, Shanghai, China; ^5^Department of Clinical Laboratory, The First Affiliated Hospital of Wenzhou Medical University, Zhejiang, China; ^6^Department of Gynecology and Obstetrics, Changning Maternity and Infant Health Hospital, Shanghai, China; ^7^Department of Gynecology and Obstetrics, The First Affiliated Hospital of University of South China, Hengyang, China; ^8^Department of Clinical Laboratory, Suzhou Municipal Hospital, Suzhou, China; ^9^Department of Cancer Epidemiology, National Cancer Center/National Clinical Research Center for Cancer/Cancer Hospital, Chinese Academy of Medical Sciences and Peking Union Medical College, Beijing, China; ^10^Center for Primary Health Care Research, Lund University, Lund, Sweden; ^11^Institute of Cancer and Basic Medicine (ICBM), Chinese Academy of Sciences (CAS), Hangzhou, China; ^12^Department of Cancer Prevention, Cancer Hospital of the University of CAS (Zhejiang Cancer Hospital), Hangzhou, China; ^13^Zhejiang Provincial Office for Cancer Prevention and Control, Zhejiang Cancer Center, Hangzhou, China

**Keywords:** human papillomavirus, cervical cancer, vaccine, prevalence, co-infection

## Abstract

**Background:** The epidemiological feature of human papillomavirus (HPV) infection is distinctive in China. We aimed to investigate the multi-infection patterns and co-infection preference of 27 HPV types among gynecological outpatients across China.

**Methods:** Overall 137,943 gynecological outpatients were recruited from eight tertiary hospitals located in seven regions of China, between July 1st, 2014 and December 31st, 2016. The overall, region-specific, age-specific and type-specific prevalence of HPV infection were calculated, respectively. The pattern of HPV infection was also evaluated. Furthermore, rate ratio was calculated to evaluate the co-infection preference of any two HPV genotypes.

**Results:** The overall prevalence of 27 HPVs' [17 high-risk (hr)/10 low-risk (lr)] infection was 23.5%. The age-specific HPV prevalence showed a “U-shaped” pattern. The most prevalent hrHPV genotypes were 16, 52, and 58. Multiple infections were detected in 25.8% of the HPV-positive women, in which dual infection was more prevalent. HPV 16/18 were likely to co-infected with HPV 31 but unlikely with HPV 52/58, i.e., the co-infection of HPV 16 with HPV 31 was high (3.5-fold), but low for HPV 58 (1.8-fold), and 52 (1.2-fold), while the co-infection of HPV 18 with HPV 31 was high (4.3-fold), but low for HPV 52 (1.9-fold), and 58 (1.7-fold).

**Conclusions:** We found age-specific prevalence of HPV infection showed a “U-shaped” pattern for high and low risk HPV, suggesting the importance of screening among younger women and the necessary of detection among older women. We found a novel co-infection preference of HPV 16/18 with 31, 52, and 58, suggesting a need of developing and marketing prophylactic HPV vaccines that protect against more genotypes in China.

## Introduction

Cervical cancer, being the 4th most frequent cancer among women, is major public health issue worldwide (1). It's estimated that more than 85% of the incident cases and deaths from cervical cancer occurred in the developing countries (2). In China, cervical cancer is the most frequent gynecological cancer (3); therefore, it is urgent to prevent and control cervical cancer burden in China.

It is well-known that persistent infection with oncogenic types of human papillomavirus (HPV) causes cervical cancer. More than 200 genotypes of HPV have been identified and among them, 13 types were classified as oncogenic (4). HPV genotypes can be further classified into high-risk HPV (hrHPV, such as HPV 16, 18, 31, 33, 35, 39, 45, 51, 52, 56, 58, 59, 66, and 68) and low-risk HPV (lrHPV, such as HPV 6, 11, 40, 42, 43, and 44), according to their associations with cervical cancer (5, 6). HPV 16 (belongs to α9 species) and 18 (α7 species), being the most prevalent hrHPV genotypes, cause ~70% of invasive cervical cancers (ICC) globally, while the lrHPV genotypes such as HPV 6 and 11 (α10 species) are associated with hyperplastic lesions (7). Additionally, co-infection with multiple genotypes is commonly detected among HPV-positive individuals (8). Also, it has been reported that individuals infected with one genotype had the tendency to harbor additional genotypes due to sexual transmission of genital HPV infections (9, 10). However, it remains unclear whether specific combination of genotypes for the co-infections is prevalent. In fact, this is important for the evaluation of effects of prophylactic HPV vaccines which could prevent the infection of certain HPV genotypes.

Prophylactic HPV vaccine is the best approach for prevention and control of cervical cancer burden, which is widely used in more than 160 countries (11). Currently, three types of vaccines are available on the market, including bivalent vaccine (targeting HPV 16 and 18), quadruple vaccine (protecting against HPV 16, 18, 6, and 11), and 9-valent vaccine (protecting against HPV 6, 11, 16, 18, 31, 33, 45, 52, and 58). The infection rates of vaccine-targeted HPV genotypes (e.g., 16 and 18) are drastically declined after vaccination (12, 13). While partial cross-protection of vaccine against HPV 31, 33, and 45 has been reported (14–17), increased infection rate of non-vaccine genotypes (such as HPV 52) was also found (14, 15). Bivalent HPV 16/18 vaccine was firstly introduced to China in 2016, which was delayed more than 10 years compared to Europe and northern America. Subsequently, quadruple vaccine and 9-valent vaccine entered on the market in China. However, all available prophylactic vaccines on Chinese market were invented using epidemiological data only from western countries. In fact, the prevalence of HPV infection and genotype distribution vary between countries and regions (18). For instance, some hrHPV genotypes (in addition to HPV 16 and 18), particularly HPV 52 and 58, were much more common in Asia (e.g., China), compared to other regions (19).

To the best of our knowledge, investigations on the co-infection preference of specific genotypes of HPV in Chinese population are scarce. Updated information on the prevalence and distribution of type-specific HPV infection, and multi-infection patterns among Chinese population is highly warranted for the choice of appropriate HPV vaccines, and timely assessment of vaccine efficacy and cost-effectiveness. Therefore, we aimed to investigate the prevalence and distribution of type-specific HPV infection among gynecological outpatients across China and to further evaluate the multi-infection patterns and co-infection preference of 27 HPV genotypes.

## Materials and Methods

### Study Population

We only recruited gynecological outpatients who attend tertiary hospitals across China. Eight tertiary hospitals from seven regions of China, including four regions (Beijing, Shanghai, Jiangsu, and Zhejiang) located in Eastern China, one region (Hunan) in Central China and two regions (Shaanxi, Sichuan) in Western China (Figure S1), participated in this study. Overall 137,943 gynecological outpatients were enrolled in this study between July 1st, 2014 and December 31st, 2016. This study was approved by the Ethics Committee of West China Second University Hospital (2017026) in accordance with the Ethical Principles for Biomedical Research Involving Human Subjects (Ministry of Health of the People's Republic of China) and with the Declaration of Helsinki for Human Research of 1974 (last modified in 2000). Written informed consent was obtained from all participants.

### Specimen Collection

Cervical exfoliated cell samples were collected from all participants by gynecologists according to the standard operation procedure. Cell samples were collected in 2.5 ml of cell preserve solution (Tellgen Corporation, Shanghai, China) for HPV DNA testing.

### HPV Genotyping

The Tellgenplex™ HPV DNA Test was performed using a Luminex-based suspension beads array to identify HPV types. The experimental protocol includes DNA extraction, PCR amplification, bead-coated hybridization, and digital signal processing. The Tellgenplex™ HPV DNA Test can identify 27 HPV types, including 14 hrHPVs (HPV 16, 18, 31, 33, 35, 39, 45, 51, 52, 56, 58, 59, 66, and 68), three potential high-risk HPVs (phrHPVs: HPV 26, 53, and 82) and 10 lrHPVs (HPV 6, 11, 40, 42, 43, 44, 55, 61, 81, and 83; Tellgen Corporation, Shanghai, China).

### Statistical Analysis

We calculated overall, region-specific and age-specific prevalence of HPV infection, respectively. Difference in the prevalence of HPV infection between each age group (≤19, 20–29, 30–39, 40–49, 50–59, and ≥60 years) was analyzed using Chi-square test or Fisher's exact test. Subsequently, the prevalence and distribution of type-specific HPV infection were calculated and, further analyses were stratified by region and infection pattern [single, dual and multiple infection (≥3)]. Furthermore, the infection pattern of 27 HPV types was evaluated, i.e., the prevalence of single, dual and multiple infection was calculated, respectively. The age-specific infection pattern of 27 HPV types was also evaluated. Moreover, the number of co-infections of 14 hrHPVs (HPV 16, 18, 31, 33, 35, 39, 45, 51, 52, 56, 58, 59, 66, and 68) and 2 lrHPVs (HPV 6 and 11) was calculated, respectively. Finally, in order to further investigate the co-infection preference of any two HPV types (such as HPV A and HPV B), we selected the aforementioned HPV types (i.e., 14 hrHPVs and 2 lrHPVs) to calculate the rate ratio, using the real infection rate of HPV A/B as the numerator and their theoretical infection rates (multiplication of the single infection rate of HPV A and HPV B) as the denominator.

All statistical analyses were performed using the SAS statistical software, version 9.4 (SAS Institute Inc., Cary, NC, USA). Graphs were generated using R version 3.4.2 (R Foundation for Statistical Computing, Vienna, Austria). Two-sided *P* < 0.05 was considered statistically significant.

## Results

### Overall and Region-Specific Prevalence of HPV Infection

Overall 137,943 gynecological outpatients were included in the analyses (median age: 40.3 ± 10.6 years; range: 15–93 years) (Table 1). The overall prevalence rate of 27 HPVs infection (17 hrHPVs/10 lrHPVs) was 23.5% and the prevalence rate varied by regions, e.g., Shaanxi/Jiangsu had the highest prevalence (26.0%), while Beijing (19.4%) had the lowest prevalence rate. Overall prevalence rate for 17 hrHPVs reached 19.4% (Shaanxi with 22.2% ranked as Top 1 and Beijing with 15.4% as the last), while prevalence rate for 10 lrHPVs reached 7.0% (Jiangsu with 10.9% ranked as Top 1 and Zhejiang with 6.3% as the last) (Table 2).

**Table 1:** Basic character of the study population (*n* = 137,943).

**Character**	**Number**	**Percentage (%)**
**Age (years)**		
Median age	40.3 ± 10.6	–
**Age group (years)**		
≤ 19	463	0.3
20–29	23,449	17.0
30–39	43,267	31.4
40–49	45,589	33.1
50–59	18,432	13.4
≥60	6,743	4.8
**HPV genotype**		
17 hrHPVs	26,711	82.3
10 lrHPVs	9,664	29.8
27 HPVs	32,469	–
**Infection pattern**				
Single infection	24,089	74.2
Dual infection	6,106	18.8
Multiple infection (≥3)	2,274	7.0

*hrHPV, high-risk HPV; lrHPV, low-risk HPV*.

**Table 2:** Overall and region-specific prevalence of 27 HPV genotypes.

**Region**	**Sample number**	**Infection rate**						
		**17 hrHPVs (%)**	**14 hrHPVs (%)**	**3 phrHPVs (%)**	**10 lrHPVs (%)**	**2 lrHPVs (%)**	**Other 8 lrHPVs (%)**	**27 HPVs (%)**
Beijing	10,374	15.4	14.2	2.2	6.7	1.3	5.6	19.4
Shaanxi	29,999	22.2	20.5	2.9	6.9	1.3	5.9	26.0
Sichuan	25,254	20.9	19.0	2.9	6.7	1.3	5.5	25.1
Shanghai	13,885	20.9	19.3	3.1	8.5	1.5	7.3	25.4
Jiangsu	4,908	19.6	18.1	2.8	10.9	2.3	9.1	26.0
Zhejiang	42,405	16.6	14.8	2.6	6.3	1.1	5.4	20.7
Hunan	11,118	20.3	18.8	2.5	7.5	2.3	5.5	24.8
Total	13,7943	19.4	17.7	2.8	7.0	1.4	5.9	23.5

*hrHPV, high-risk HPV; phrHPV, potential high-risk HPV; lrHPV, low-risk HPV*.

*14 hrHPVs: HPV 16, 18, 31, 33, 35, 39, 45, 51, 52, 56, 58, 59, 66 and 68*.

*3 phrHPVs: HPV 26, 53 and 82*.

*2 lrHPVs: HPV 6 and 11*.

*Other 8 lrHPVs: HPV 40, 42, 43, 44, 55, 61, 81 and 83*.

### Age-Specific Prevalence of HPV Infection

**Figure 1:**
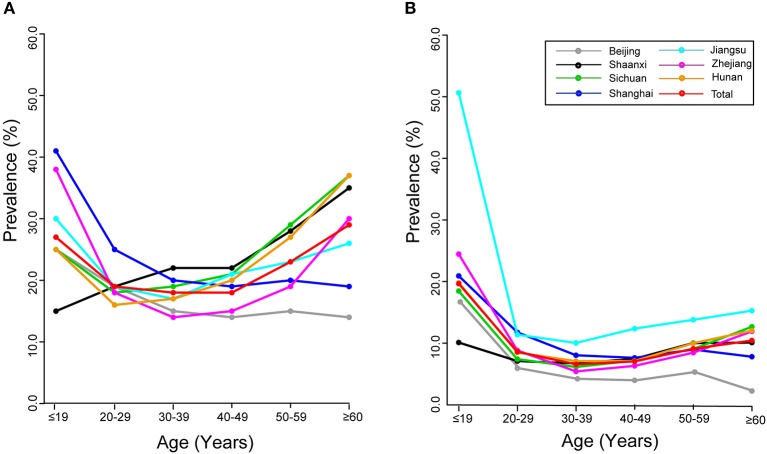
Age-specific prevalence of HPV infection: overall and by region. **(A)** 17 hrHPVs. **(B)** 10 lrHPVs.

Overall age-specific prevalence of HPV infection showed a “U-shaped” pattern (Figures 1A,B). Two peaks for age groups were observed for 17 hrHPVs (26.6% for age ≤19 years and 28.9% for age ≥60 years; Figure 1A) and for 10 lrHPVs (19.0% for age ≤19 years and 10.0% for age ≥60 years; Figure 1B). Notably, the prevalence rate of hrHPV infection among women aged ≤19 years in Shanghai reached 40.5%, while the prevalence rate of lrHPV infection among women aged ≤19 years in Jiangsu were extremely high (50.0%) (Table S1, Figures 1A,B).

### Prevalence of Type-Specific HPV Infection

**Figure 2:**
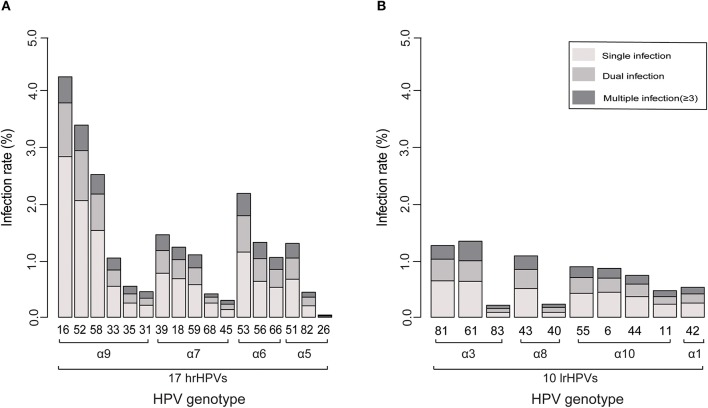
Ranking of the prevalence of each genotype related to its phylogenetic group. **(A)** 17 hrHPVs. **(B)** 10 lrHPVs.

Type-specific HPV infection rate is presented in Figure 2A (hrHPVs) and Figure 2B (lrHPVs). The most prevalent hrHPV genotype was HPV 16 (infection rate: 4.4%), followed by HPV 52 (3.5%) and 58 (2.6%) and among all HPV-positive women, the proportion reached 13.9, 11.1, and 8.3%, respectively. For the lrHPVs, the most commonly detected genotypes were HPV 81 and 61 (α3 species; infection rate: both 1.3%), accounting for 4.1% of the total HPV-positive women, respectively.

Variations by regions were found (Tables S2, S3). For each region, HPV 16, 52 and 58 were most prevalent genotypes. For instance, HPV 52 as the leading genotype was found in Beijing (with infection rate 3.0%), Jiangsu (4.0%), and Zhejiang (3.6%), while HPV 16 was found as most common genotype for other regions. Moreover, the infection rates of HPV 6 and 11 were higher in Jiangsu (1.24 and 1.12%, respectively) and Hunan (1.57 and 0.72%, respectively) compared to other regions.

### Distribution of Multiple Infections

**Figure 3:**
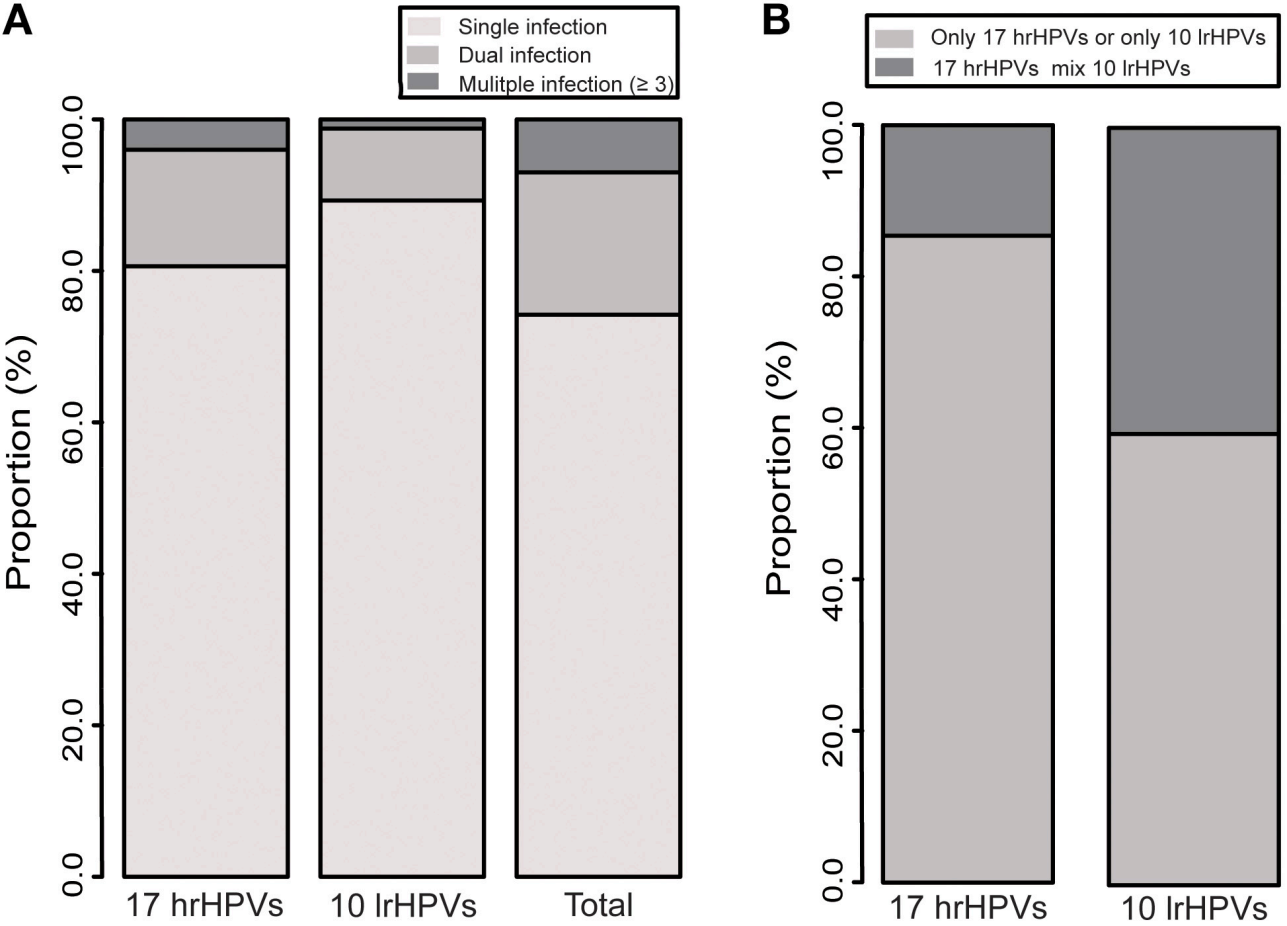
**(A)** Distribution of multiple infections of 27 HPV genotypes. **(B)** Co-infection of 17 hrHPVs and 10 lrHPVs.

The prevalence of multiple infections is shown in Figures 3A,B and Table S4. Single infection was found in 74.2% gynecological outpatients, accounting for 80.6% for hrHPVs group and 89.3% for lrHPVs group. Overall 25.8% outpatients were found with multiple infections and 72.9% with dual infection. Among the multiple infections, 14.6% of the hrHPVs co-infected with lrHPVs were detected whereas 40.4% of the lrHPVs co-infected with hrHPVs.

In the single infection group, HPV 16 was the most prevalent genotype (with infection rate 3.0%), followed by HPV 52 (2.2%) and 58 (1.6%) (Table S3). In the dual infection group, the most commonly detected mixed genotypes were HPV 16+58 (α9 species; 283 cases), followed by HPV 16+52 (α9 species; 265), 52+58 (α9 species; 242), and 16+18 (α9 and α7 species; 195) (Table S5).

Age-specific multiple infections of 27 HPV genotypes are shown in Table S6. The highest proportion of single infection was found among women aged 40–49 years, accounting for 77.8% of all HPV-positive women. However, dual infection and multiple infection were more frequently found among women aged ≤19 years, with the proportion of 22.4 and 17.0%, respectively. Multiple infection was more common among women aged ≤19 years for hrHPV and lrHPV groups.

### Co-infection Preference

**Figure 4:**
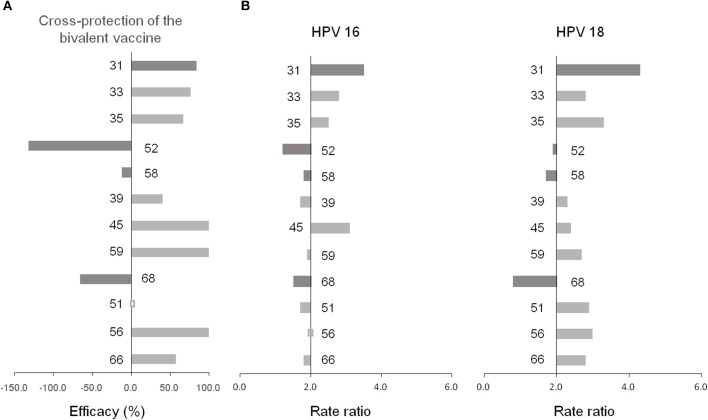
**(A)** Cross-protection of the bivalent vaccine (14). **(B)** Co-infection preference of 14 hrHPVs.

The co-infection of any two genotypes is presented in Figure 4 and Table S7. The co-infection of HPV 16 with one of hrHPVs reached more than two-fold elevated for HPV 31 (3.5), 45 (3.1), 33 (2.8), 18 (2.5), and 35 (2.5), while the co-infection of HPV 18 with one of hrHPVs reached more than two-fold elevated for HPV 31 (4.3), 35 (3.3), 56 (3.0), 51 (2.9), 33 (2.8), 66 (2.8), 59 (2.7), 16 (2.5), 45 (2.4), and 39 (2.3). Interestingly, the co-infection of HPV 18 with 68 reached only 0.8-fold. Furthermore, the co-infection of HPV 6 with one of hrHPVs reached more than two-fold elevated for HPV 59 (4.3), 51 (3.8), 11 (3.5), 66 (3.4), 39 (3.2), 35 (3.0), 18 (2.8), 33 (2.7), 56 (2.5), 45 (2.4), 16 (2.3), and 52 (2.1). Additionally, the co-infection of HPV 11 with one of hrHPVs reached more than two-fold elevated for HPV 45 (5.4), 59 (4.9), 31 (4.3), 56 (4.2), 35 (3.9), 6 (3.5), 18 (3.4), 51 (3.3), 39 (2.9), 66 (2.8), 68 (2.4), and 52 (2.1).

## Discussion

We investigated, for the first time systematically in China, the prevalence and distribution of type-specific HPV infection among gynecological outpatients, and also the multi-infection patterns and co-infection preference of 27 HPV types, using data from 137,943 Chinese gynecological outpatients across seven regions of China. We found the overall prevalence of HPV infection (27 HPVs) reached 23.5%, with 19.4% for hrHPV group and 7.0% for lrHPV group. The age-specific prevalence of HPV infection showed a “U-shaped” pattern for hrHPV and lrHPV, with two peaks for age ≤19 years and for age ≥60 years (26.6 and 28.9% for hrHPV; 19.0 and 10.0% for lrHPV, respectively). The overall prevalence of hrHPV infection was 19.4% and the most prevalent hrHPV genotypes were HPV 16 (4.4%), followed by HPV 52 (3.5%) and 58 (2.6%). Multiple infections were found in 25.8% of HPV-positive women, in which dual infection was more prevalent. HPV 16/18 were likely to co-infected with HPV 31 (the co-infection of HPV 16 with HPV 31 was 3.5-fold elevated and the co-infection of HPV 18 with HPV 31 was 4.3-fold elevated) but unlikely with HPV 52/58.

Our finding of overall prevalence rate of hrHPV infection (19.4%) was slightly lower compared to a pooled analysis (23.2%) (20), but much higher than a pooled analysis including 17 population-based studies from nine regions across China (11.2%) (21), in line with previous population-based investigations reported that the prevalence of hrHPV varied by regions in China (range: 9.9–27.5%), with highest prevalence in Shaanxi and lowest in Beijing (2, 22). This disparity may be attributed to different region and target population, time period, and lab methodology for HPV genotyping. In our study, the fluctuation of region-specific prevalence of hrHPV may also be influenced by different economic conditions, lifestyles as well as the knowledge and awareness about HPV vaccination (23, 24).

Our finding of a “U-shaped” pattern of age-specific prevalence for hrHPV and lrHPV group (with two peaks for young women aged ≤19 years and for older women aged ≥60 years) is generally consistent with previous similar investigations in China (2, 25–28), but differs from the pattern observed in the American women, e.g., the HPV prevalence peaked only in the age group of mid-twenties and then steadily declined with the increase of age (29). Our finding of “two peak” pattern may be partly attributed to the natural history of HPV infection (30). Younger women are sensitive to HPV infection after the sexual activity due to the immature immune protection (27), while second peak of HPV prevalence in older women may be attributed to the persistent HPV infection or reactivation of a latent HPV infection, which was associated with physiological and immunological disorders resulting from hormone fluctuations during their menopausal transition (31).

We found that HPV 16, 52, and 58 were the predominant genotypes, in line with previous investigations in China (25, 27, 32–35). Similarly, HPV 16 is reported to be prevalent worldwide (18, 24), while HPV 52 and 58 are more common in the Asian population (36). Surprisingly, HPV 18 [very common in western women (18)] was only the 8th most common genotype in our samples.

We found lrHPVs were also detectable in the cervical exfoliated cell samples, indicating the necessary of detecting hrHPVs and lrHPVs at the same time, particularly 14 hrHPVs with 2 lrHPVs (HPV 6 and 11). The prevalent genotypes found in our study suggested that prophylactic HPV vaccines protecting against additional HPV genotypes (especially 52 and 58) may offer a better protection efficacy for Chinese and other Asian populations.

We found that single infection was more common among the HPV-positive women and multiple infections accounted for a small proportion (25.8%), consistent with previous investigations in Chinese population (27, 37–39) and with studies in other populations (9, 10). We found the most prevalent mixed genotypes were HPV 16+58, 16+52 and 52+58 (all belong to α9 species), in line with previous studies from China (27), but different from Chaturvedi et al. (10) study found that some genotype combinations (e.g., HPV 31+33 and 45+68; also belonging to α9 and α7 species) were more likely to be involved in the co-infections. These findings suggest that phylogenetically-related HPV genotypes have the tendency to cluster together, yet the mechanisms remain unknown.

There is no consensus on the association of multiple infections with occurrence or progression of cervical cancer. For instance, Fife et al. (40) showed that multiple hrHPV infection tended to increase the risk of cervical diseases, while Jung et al. (41) found that multiple infections were less frequently associated with the cervical neoplasia. We found that multiple infections are more common among younger women, which is in line with previous studies (42, 43) and suggests more sexual activity of younger women may lead to the sexual transmission of multiple HPV genotypes.

We found that HPV 31 was most likely to co-infected with HPV 16/18, consistent with the cross-protective efficacy of bivalent vaccine against HPV 31 and with findings in other populations (44, 45). We found that some genotypes (HPV 68, 52 and 58, particularly 68) were less likely to co-infected with HPV 16/18, in line with the studies by Wheeler et al. (14) and Tabrizi et al. (15) in Australia, but not with the study by Woestenberg et al. (45). The immune mechanisms of vaccine-induced cross-protection against non-vaccine genotypes are not fully understood yet, which may be related to the conserved aminoacid sequences or structural similarities within shared neutralizing epitopes among HPV genotypes (46, 47).

Our study has a number of strengthens and limitations. Firstly, we systematically investigated multi-infection patterns and co-infection preference of 27 HPV types (17 hrHPVs/10 lrHPVs), using data from 137,943 Chinese gynecological outpatients across seven regions of China. Secondly, our study focused on gynecological outpatients who were identified as a high-risk population of HPV infection, which could help the clinicians to better understand the prevalence and distribution of type-specific HPV infection. Thirdly, the association between the co-infection preference and cross-protective efficacy of HPV vaccine was revealed for the first time, which might help to better understand the mechanisms of cross-protection of vaccines and to provide guidance on HPV vaccine development and application in the future. Major limitation concerns sample source. Subjects in the youngest (0.3%) and oldest (4.8%) age group accounted for a small proportion, which might over-estimate age-specific prevalence of HPV infection.

## Conclusions

In summary, using data from 137,943 Chinese gynecological outpatients across China, we found the overall prevalence of HPV infection reached 23.5% and genotype distribution varied by regions, suggesting the prevention of HPV infection in a region-specific pattern. We found age-specific prevalence of HPV infection showed a “U-shaped” pattern for hrHPV and lrHPV (with two peaks observed in younger and older women), suggesting the importance of screening among younger women and the necessary of HPV detection among older women. We found that a novel co-infection preference of HPV 16/18 with 31, 52, and 58, suggesting vaccines could also protect against HPV 52/58 are highly warranted in China. Our findings may help to reveal the mechanisms of cross-protection of HPV vaccines and to indicate the necessary of developing and marketing prophylactic HPV vaccines that protect against more genotypes (particularly HPV 52 and 58), which may offer better protection efficacy for Chinese population.

## Data Availability Statement

The original contributions presented in the study are included in the article/Supplementary Material, further inquiries can be directed to the corresponding author.

## Ethics Statement

The studies involving human participants were reviewed and approved by Ethics Committee of West China Second University Hospital. Written informed consent to participate in this study was provided by the participants' legal guardian/next of kin.

## Author Contributions

MX and TC were responsible for the study concept and design. TC, GL, and XJ obtained funding. ZW, HZ, YM, WX, HX, and WC acquired data. GL, XJ, BS, HT and JJ analyzed and interpreted data. GL, XJ, BS, and TC drafted the manuscript, and all authors revised it for important intellectual content. TC and MX are the guarantors of this work.

### Conflict of Interest

BS was employed by the company Tellgen Corporation, Shanghai, China. The remaining authors declare that the research was conducted in the absence of any commercial or financial relationships that could be construed as a potential conflict of interest.

## Supplementary Material

Figure S1 National map of China showing all the geographical sites included in this study.
