# Supplementary figures and images for: Multi-Infection Patterns and Co-infection Preference of 27 Human Papillomavirus Types Among 137,943 Gynecological Outpatients Across China
Source: Front Oncol. 2020 Apr 7;10:449. doi: 10.3389/fonc.2020.00449 (PMC7154087; doi:10.3389/fonc.2020.00449)

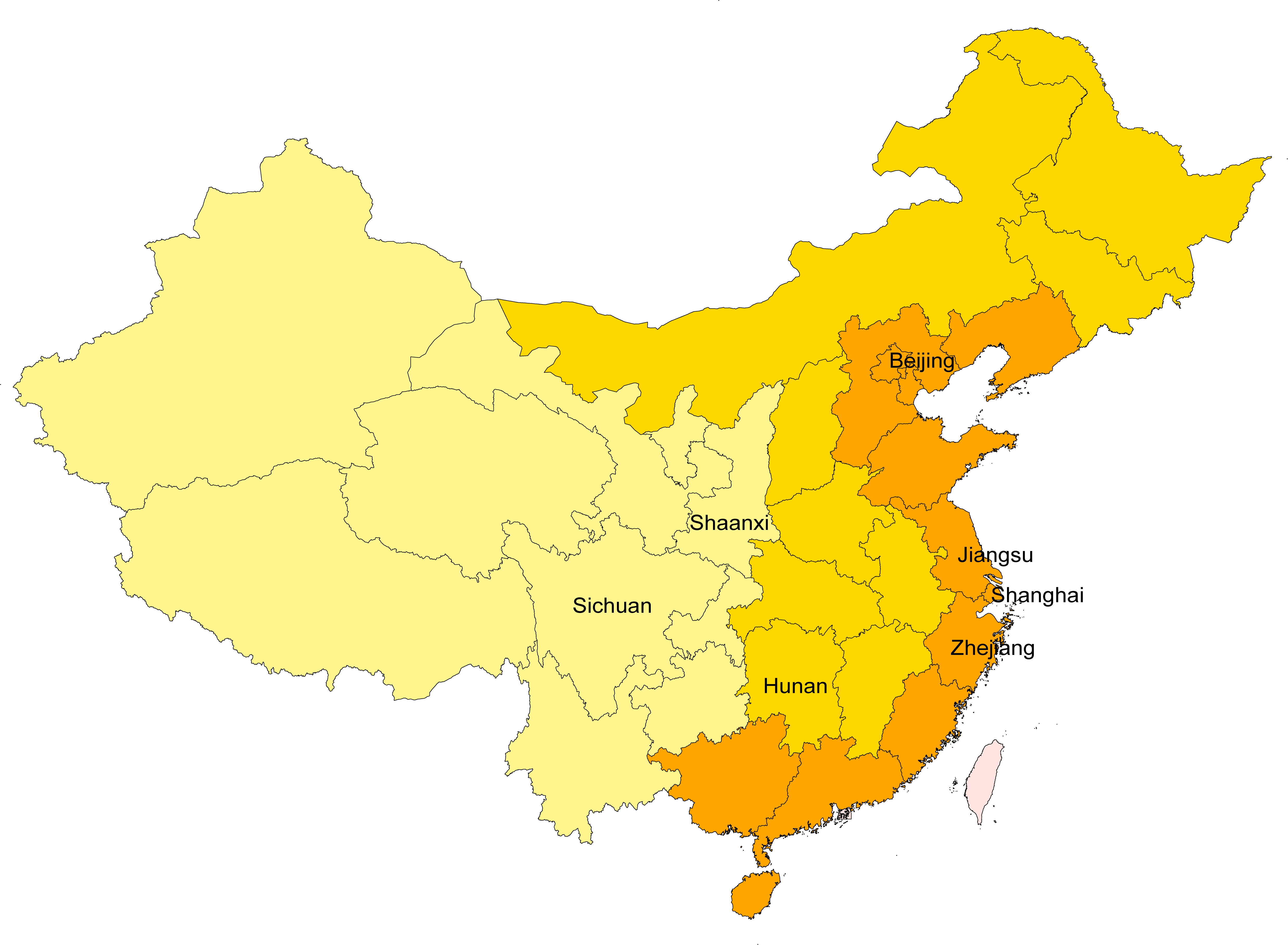

Supplement: Figure S1 — National map of China showing all the geographical sites included in this study. [file Image_1.TIF]
