# Supplementary material for: Multi-Infection Patterns and Co-infection Preference of 27 Human Papillomavirus Types Among 137,943 Gynecological Outpatients Across China
Source: Front Oncol. 2020 Apr 7;10:449. doi: 10.3389/fonc.2020.00449 (PMC7154087; doi:10.3389/fonc.2020.00449)
Supplement: Supplementary file 2 [file Table_1.DOC]

**Table S1 Age-specific prevalence of 17 hrHPVs and 10 lrHPVs infection:** overall and by region

| **Agegroup** |  |  |  | **Region** |  |  |  |  |
| --- | --- | --- | --- | --- | --- | --- | --- | --- |
|  | **Beijing** | **Shaanxi** | **Sichuan** | **Shanghai** | **Jiangsu** | **Zhejiang** | **Hunan** | **Total** |
| **17 hrHPVs** |  |  |  |  |  |  |  |  |
| ≤19 | 25.0% | 14.9% | 24.8% | 40.5% | 30.0% | 38.1% | 24.8% | 26.6% |
| 20-29 | 18.7% | 18.7% | 18.4% | 24.7% | 19.4% | 18.5% | 15.9% | 19.3% |
| 30-39 | 15.0% | 21.5% | 18.7% | 19.5% | 17.3% | 14.0% | 17.1% | 17.6% |
| 40-49 | 13.6% | 21.5% | 21.3% | 19.1% | 21.2% | 15.1% | 19.6% | 18.3% |
| 50-59 | 15.2% | 27.5% | 28.8% | 20.0% | 23.4% | 19.1% | 27.2% | 22.5% |
| ≥60 | 13.9% | 34.6% | 36.9% | 19.4% | 26.5% | 30.5% | 36.9% | 28.9% |
| **10 lrHPVs** |  |  |  |  |  |  |  |  |
| ≤19 | 18.8% | 9.5% | 17.8% | 20.3% | 50.0% | 23.8% | 19.2% | 19.0% |
| 20-29 | 8.0% | 6.5% | 6.8% | 11.1% | 10.7% | 8.2% | 7.8% | 8.0% |
| 30-39 | 6.3% | 6.1% | 5.5% | 7.4% | 9.4% | 4.8% | 6.4% | 5.9% |
| 40-49 | 6.0% | 6.8% | 6.5% | 7.0% | 11.7% | 5.7% | 6.5% | 6.4% |
| 50-59 | 7.4% | 9.4% | 8.4% | 8.3% | 13.2% | 7.8% | 9.3% | 8.5% |
| ≥60 | 4.5% | 9.7% | 12.3% | 7.4% | 14.9% | 11.5% | 11.6% | 10.0% |

hrHPV: high-risk HPV; lrHPV: low-risk HPV

17 hrHPVs: HPV 16, 18, 26, 31, 33, 35, 39, 45, 51, 52, 53, 56, 58, 59, 66, 68 and 82

10 lrHPVs: HPV 6, 11, 40, 42, 43, 44, 55, 61, 81 and 8

**Table S2 Prevalence of type-specific HPV infection: overall and by region**

| **Phylogenetic group** | **HPV genotype** | **Region** | | | | | | | |
| --- | --- | --- | --- | --- | --- | --- | --- | --- | --- |
|  |  | **Total** | **Beijing** | **Shaanxi** | **Sichuan** | **Shanghai** | **Jiangsu** | **Zhejiang** | **Hunan** |
| α9 | 16 | 4.43% | 2.93% | 6.22% | 5.37% | 4.24% | 3.77% | 2.79% | 5.68% |
|  | 52 | 3.54% | 3.04% | 2.93% | 4.02% | 3.68% | 3.95% | 3.57% | 4.07% |
|  | 58 | 2.64% | 1.83% | 2.97% | 2.91% | 3.09% | 2.42% | 2.34% | 2.55% |
|  | 33 | 1.09% | 0.81% | 1.05% | 1.00% | 1.28% | 1.39% | 1.04% | 1.48% |
|  | 35 | 0.57% | 0.52% | 0.61% | 0.46% | 0.84% | 0.67% | 0.52% | 0.61% |
|  | 31 | 0.48% | 0.47% | 0.66% | 0.39% | 0.58% | 0.61% | 0.29% | 0.74% |
|  |  |  |  |  |  |  |  |  |  |
| α7 | 39 | 1.53% | 1.20% | 1.68% | 1.47% | 1.79% | 1.69% | 1.39% | 1.73% |
|  | 18 | 1.30% | 0.90% | 1.68% | 1.23% | 1.58% | 1.28% | 1.05% | 1.41% |
|  | 59 | 1.15% | 0.92% | 1.39% | 1.28% | 1.64% | 1.10% | 0.85% | 1.03% |
|  | 68 | 0.44% | 0.40% | 0.56% | 0.39% | 0.58% | 0.55% | 0.35% | 0.34% |
|  | 45 | 0.30% | 0.23% | 0.36% | 0.26% | 0.36% | 0.24% | 0.30% | 0.30% |
|  |  |  |  |  |  |  |  |  |  |
| α6 | 53 | 2.28% | 1.84% | 2.24% | 2.55% | 2.31% | 2.18% | 2.29% | 2.13% |
|  | 56 | 1.38% | 1.60% | 1.80% | 1.33% | 1.77% | 1.41% | 1.02% | 1.00% |
|  | 66 | 1.10% | 1.23% | 1.48% | 0.95% | 1.40% | 1.20% | 0.77% | 1.09% |
|  |  |  |  |  |  |  |  |  |  |
| α5 | 51 | 1.37% | 1.23% | 1.65% | 1.35% | 1.84% | 1.96% | 1.04% | 1.06% |
|  | 82 | 0.46% | 0.44% | 0.65% | 0.35% | 0.73% | 0.59% | 0.32% | 0.40% |
|  | 26 | 0.04% | 0.02% | 0.02% | 0.04% | 0.09% | 0.06% | 0.03% | 0.06% |
|  |  |  |  |  |  |  |  |  |  |
| α3 | 81 | 1.32% | 1.11% | 1.01% | 1.33% | 1.68% | 2.47% | 1.29% | 1.51% |
|  | 61 | 1.30% | 1.23% | 1.44% | 1.05% | 1.61% | 1.61% | 1.30% | 0.98% |
|  | 83 | 0.22% | 0.40% | 0.15% | 0.19% | 0.40% | 0.26% | 0.17% | 0.22% |
|  |  |  |  |  |  |  |  |  |  |
| α8 | 43 | 1.13% | 0.95% | 1.24% | 1.17% | 1.47% | 2.04% | 0.88% | 1.08% |
|  | 40 | 0.24% | 0.29% | 0.32% | 0.24% | 0.32% | 0.53% | 0.14% | 0.20% |
|  |  |  |  |  |  |  |  |  |  |
| α10 | 55 | 0.93% | 1.10% | 1.04% | 0.83% | 1.04% | 1.39% | 0.78% | 0.91% |
|  | 6 | 0.90% | 0.88% | 0.89% | 0.80% | 1.04% | 1.24% | 0.71% | 1.57% |
|  | 44 | 0.77% | 0.66% | 0.44% | 0.80% | 1.04% | 1.24% | 0.83% | 0.88% |
|  | 11 | 0.49% | 0.42% | 0.41% | 0.52% | 0.51% | 1.12% | 0.40% | 0.72% |
|  |  |  |  |  |  |  |  |  |  |
| α1 | 42 | 0.55% | 0.67% | 0.81% | 0.38% | 0.71% | 0.79% | 0.42% | 0.33% |

**Table S3 Prevalence of type-specific HPV infection: by infection pattern**

| **Phylogenetic group** | **HPV genotype** | **Proportion** | **Infection pattern** | | |
| --- | --- | --- | --- | --- | --- |
|  |  |  | **Single infection** | **Dual infection** | **Multiple infection (≥3)** |
| α9 | 16 | 13.90% | 2.96% | 0.99% | 0.48% |
|  | 52 | 11.07% | 2.15% | 0.92% | 0.47% |
|  | 58 | 8.26% | 1.60% | 0.67% | 0.36% |
|  | 33 | 3.40% | 0.57% | 0.30% | 0.22% |
|  | 35 | 1.79% | 0.26% | 0.17% | 0.14% |
|  | 31 | 1.50% | 0.22% | 0.13% | 0.12% |
|  |  |  |  |  |  |
| α7 | 39 | 4.79% | 0.81% | 0.42% | 0.29% |
|  | 18 | 4.07% | 0.71% | 0.35% | 0.23% |
|  | 59 | 3.61% | 0.60% | 0.31% | 0.24% |
|  | 68 | 1.37% | 0.26% | 0.11% | 0.06% |
|  | 45 | 0.95% | 0.14% | 0.10% | 0.07% |
|  |  |  |  |  |  |
| α6 | 53 | 7.13% | 1.20% | 0.67% | 0.41% |
|  | 56 | 4.31% | 0.66% | 0.42% | 0.30% |
|  | 66 | 3.44% | 0.55% | 0.33% | 0.22% |
|  |  |  |  |  |  |
| α5 | 51 | 4.28% | 0.70% | 0.39% | 0.27% |
|  | 82 | 1.45% | 0.21% | 0.16% | 0.09% |
|  | 26 | 0.12% | 0.02% | 0.01% | 0.01% |
|  |  |  |  |  |  |
| α3 | 81 | 4.14% | 0.67% | 0.40% | 0.25% |
|  | 61 | 4.06% | 0.66% | 0.38% | 0.36% |
|  | 83 | 0.68% | 0.09% | 0.07% | 0.06% |
|  |  |  |  |  |  |
| α8 | 43 | 3.54% | 0.53% | 0.35% | 0.25% |
|  | 40 | 0.76% | 0.09% | 0.09% | 0.06% |
|  |  |  |  |  |  |
| α10 | 55 | 2.91% | 0.44% | 0.29% | 0.20% |
|  | 6 | 2.82% | 0.46% | 0.26% | 0.18% |
|  | 44 | 2.40% | 0.38% | 0.23% | 0.16% |
|  | 11 | 1.53% | 0.24% | 0.14% | 0.11% |
|  |  |  |  |  |  |
| α1 | 42 | 1.72% | 0.26% | 0.17% | 0.12% |

**Table S4 Prevalence of** multiple infections of 27 HPV genotypes

|  |  | **Single infection** | |  | **Dual infection** | |  | **Multiple infection (≥3)** | |
| --- | --- | --- | --- | --- | --- | --- | --- | --- | --- |
|  |  | Infection rate | Proportion |  | Infection rate | Proportion | | Infection rate | Proportion |
| 17 hrHPVs | | 15.6% | 80.6% |  | 3.0% | 15.4% |  | 0.8% | 4.0% |
| 10 lrHPVs | | 6.3% | 89.3% |  | 0.7% | 9.5% |  | 0.1% | 1.2% |
| Total |  | 17.5% | 74.2% |  | 4.4% | 18.8% |  | 1.7% | 7.0% |

hrHPV: high-risk HPV; lrHPV: low-risk HPV

17 hrHPVs: HPV 16, 18, 26, 31, 33, 35, 39, 45, 51, 52, 53, 56, 58, 59, 66, 68 and 82

10 lrHPVs: HPV 6, 11, 40, 42, 43, 44, 55, 61, 81 and 83

**Table S5 Number of co-infections of 14 hrHPVs and 2 lrHPVs**

| **HPV genotype** | **6** | **11** | **16** | **18** | **31** | **33** | **35** | **39** | **45** | **51** | **52** | **56** | **58** | **59** | **66** | **68** |
| --- | --- | --- | --- | --- | --- | --- | --- | --- | --- | --- | --- | --- | --- | --- | --- | --- |
| **6** | 1241 | 21 | 125 | 45 | 10 | 37 | 21 | 60 | 9 | 65 | 91 | 43 | 54 | 62 | 46 | 11 |
| **11** | 21 | 674 | 55 | 30 | 14 | 15 | 15 | 30 | 11 | 30 | 49 | 39 | 23 | 38 | 21 | 7 |
| **16** | 125 | 55 | 6115 | 195 | 103 | 186 | 87 | 161 | 57 | 142 | 265 | 170 | 283 | 131 | 118 | 39 |
| **18** | 45 | 30 | 195 | 1793 | 37 | 55 | 34 | 64 | 13 | 71 | 118 | 73 | 82 | 55 | 55 | 6 |
| **31** | 10 | 14 | 103 | 37 | 659 | 64 | 32 | 22 | 9 | 26 | 41 | 17 | 42 | 16 | 13 | 8 |
| **33** | 37 | 15 | 186 | 55 | 64 | 1500 | 53 | 42 | 20 | 52 | 114 | 51 | 83 | 49 | 37 | 8 |
| **35** | 21 | 15 | 87 | 34 | 32 | 53 | 790 | 28 | 7 | 38 | 45 | 42 | 49 | 29 | 28 | 8 |
| **39** | 60 | 30 | 161 | 64 | 22 | 42 | 28 | 2112 | 10 | 95 | 184 | 92 | 103 | 82 | 77 | 17 |
| **45** | 9 | 11 | 57 | 13 | 9 | 20 | 7 | 10 | 420 | 11 | 36 | 20 | 26 | 14 | 20 | 3 |
| **51** | 65 | 30 | 142 | 71 | 26 | 52 | 38 | 95 | 11 | 1888 | 142 | 96 | 110 | 70 | 67 | 20 |
| **52** | 91 | 49 | 265 | 118 | 41 | 114 | 45 | 184 | 36 | 142 | 4877 | 162 | 242 | 117 | 109 | 39 |
| **56** | 43 | 39 | 170 | 73 | 17 | 51 | 42 | 92 | 20 | 96 | 162 | 1900 | 124 | 68 | 75 | 19 |
| **58** | 54 | 23 | 283 | 82 | 42 | 83 | 49 | 103 | 26 | 110 | 242 | 124 | 3640 | 99 | 84 | 21 |
| **59** | 62 | 38 | 131 | 55 | 16 | 49 | 29 | 82 | 14 | 70 | 117 | 68 | 99 | 1592 | 66 | 14 |
| **66** | 46 | 21 | 118 | 55 | 13 | 37 | 28 | 77 | 20 | 67 | 109 | 75 | 84 | 66 | 1515 | 13 |
| **68** | 11 | 7 | 39 | 6 | 8 | 8 | 8 | 17 | 3 | 20 | 39 | 19 | 21 | 14 | 13 | 602 |

hrHPV: high-risk HPV; lrHPV: low-risk HPV

**Table S6 Age-specific** multiple infections of 27 HPV genotypes

| **Age group** |  | **Infection pattern** |  |
| --- | --- | --- | --- |
|  | **Single infection** | **Dual infection** | **Multiple infection (≥3)** |
| **27 HPVs** |  |  |  |
| ≤19 | 60.6% | 22.4% | 17.0% |
| 20-29 | 69.1% | 21.3% | 9.6% |
| 30-39 | 76.5% | 18.4% | 5.1% |
| 40-49 | 77.8% | 16.8% | 5.4% |
| 50-59 | 73.0% | 19.2% | 7.8% |
| ≥60 | 64.9% | 22.2% | 12.9% |
| **17 hrHPVs** |  |  |  |
| ≤19 | 72.4% | 17.1% | 10.5% |
| 20-29 | 75.8% | 18.6% | 5.6% |
| 30-39 | 82.4% | 14.6% | 3.0% |
| 40-49 | 83.4% | 13.8% | 2.8% |
| 50-59 | 80.6% | 15.3% | 4.1% |
| ≥60 | 73.5% | 18.7% | 7.8% |
| **10lrHPVs** |  |  |  |
| ≤19 | 80.1% | 13.6% | 6.3% |
| 20-29 | 87.1% | 11.3% | 1.6% |
| 30-39 | 91.5% | 7.9% | 0.6% |
| 40-49 | 92.1% | 6.9% | 1.0% |
| 50-59 | 87.4% | 10.7% | 1.9% |
| ≥60 | 81.0% | 18.1% | 0.9% |

hrHPV: high-risk HPV; lrHPV: low-risk HPV

17 hrHPVs: HPV 16, 18, 26, 31, 33, 35, 39, 45, 51, 52, 53, 56, 58, 59, 66, 68 and 82

10 lrHPVs: HPV 6, 11, 40, 42, 43, 44, 55, 61, 81 and 83

**Table S7 Co-infection preference of 14 hrHPVs and 2 lrHPVs**

| **HPV genotype** | **6** | **11** | **16** | **18** | **31** | **33** | **35** | **39** | **45** | **51** | **52** | **56** | **58** | **59** | **66** | **68** |
| --- | --- | --- | --- | --- | --- | --- | --- | --- | --- | --- | --- | --- | --- | --- | --- | --- |
| **6** | 1.0 | 3.5 | 2.3 | 2.8 | 1.7 | 2.7 | 3.0 | 3.2 | 2.4 | 3.8 | 2.1 | 2.5 | 1.6 | 4.3 | 3.4 | 2.0 |
| **11** | 3.5 | 1.0 | 1.8 | 3.4 | 4.3 | 2.0 | 3.9 | 2.9 | 5.4 | 3.3 | 2.1 | 4.2 | 1.3 | 4.9 | 2.8 | 2.4 |
| **16** | 2.3 | 1.8 | 1.0 | 2.5 | 3.5 | 2.8 | 2.5 | 1.7 | 3.1 | 1.7 | 1.2 | 2.0 | 1.8 | 1.9 | 1.8 | 1.5 |
| **18** | 2.8 | 3.4 | 2.5 | 1.0 | 4.3 | 2.8 | 3.3 | 2.3 | 2.4 | 2.9 | 1.9 | 3.0 | 1.7 | 2.7 | 2.8 | 0.8 |
| **31** | 1.7 | 4.3 | 3.5^*^ | 4.3 | 1.0 | 8.9 | 8.5 | 2.2 | 4.5 | 2.9 | 1.8 | 1.9 | 2.4 | 2.1 | 1.8 | 2.8 |
| **33** | 2.7 | 2.0 | 2.8 | 2.8 | 8.9 | 1.0 | 6.2 | 1.8 | 4.4 | 2.5 | 2.2 | 2.5 | 2.1 | 2.8 | 2.3 | 1.2 |
| **35** | 3.0 | 3.9 | 2.5 | 3.3 | 8.5 | 6.2 | 1.0 | 2.3 | 2.9 | 3.5 | 1.6 | 3.9 | 2.4 | 3.2 | 3.2 | 2.3 |
| **39** | 3.2 | 2.9 | 1.7 | 2.3 | 2.2 | 1.8 | 2.3 | 1.0 | 1.6 | 3.3 | 2.5 | 3.2 | 1.9 | 3.4 | 3.3 | 1.8 |
| **45** | 2.4 | 5.4 | 3.1 | 2.4 | 4.5 | 4.4 | 2.9 | 1.6 | 1.0 | 1.9 | 2.4 | 3.5 | 2.4 | 2.9 | 4.3 | 1.6 |
| **51** | 3.8 | 3.3 | 1.7 | 2.9 | 2.9 | 2.5 | 3.5 | 3.3 | 1.9 | 1.0 | 2.1 | 3.7 | 2.2 | 3.2 | 3.2 | 2.4 |
| **52** | 2.1 | 2.1 | 1.2 | 1.9 | 1.8 | 2.2 | 1.6 | 2.5 | 2.4 | 2.1 | 1.0 | 2.4 | 1.9 | 2.1 | 2.0 | 1.8 |
| **56** | 2.5 | 4.2 | 2.0 | 3.0 | 1.9 | 2.5 | 3.9 | 3.2 | 3.5 | 3.7 | 2.4 | 1.0 | 2.5 | 3.1 | 3.6 | 2.3 |
| **58** | 1.6 | 1.3 | 1.8 | 1.7 | 2.4 | 2.1 | 2.4 | 1.9 | 2.4 | 2.2 | 1.9 | 2.5 | 1.0 | 2.4 | 2.1 | 1.3 |
| **59** | 4.3 | 4.9 | 1.9 | 2.7 | 2.1 | 2.8 | 3.2 | 3.4 | 2.9 | 3.2 | 2.1 | 3.1 | 2.4 | 1.0 | 3.8 | 2.0 |
| **66** | 3.4 | 2.8 | 1.8 | 2.8 | 1.8 | 2.3 | 3.2 | 3.3 | 4.3 | 3.2 | 2.0 | 3.6 | 2.1 | 3.8 | 1.0 | 2.0 |
| **68** | 2.0 | 2.4 | 1.5 | 0.8 | 2.8 | 1.2 | 2.3 | 1.8 | 1.6 | 2.4 | 1.8 | 2.3 | 1.3 | 2.0 | 2.0 | 1.0 |

hrHPV: high-risk HPV; lrHPV: low-risk HPV；

The number indicates the co-infection preference. For instance, ^*^ indicates that the co-infection of HPV 16 with HPV 31 was high (3.5-fold).
